# 99391 A TL1 Team Approach: The Role of Parents in Physical Activity Engagement Among Adolescents with Comorbid Asthma and Obesity

**DOI:** 10.1017/cts.2021.766

**Published:** 2021-03-30

**Authors:** Jacqlyn Yourell, Natalie Koskela-Staples, David Fedele, Jennifer Doty

**Affiliations:** University of Florida

## Abstract

ABSTRACT IMPACT: Our research highlights the need for both parental and clinical support to promote PA engagement among higher risk youth with comorbid asthma and obesity; these findings will inform research and clinical efforts in the youth development, prevention science, and clinical psychology fields. OBJECTIVES/GOALS: Asthma incidence doubles in youth with obesity. Physical activity (PA) is beneficial for asthma management; however, parental influence on PA levels among youth with asthma and obesity is poorly understood. This study examines the association of parents and PA among youth with asthma and/or obesity, accounting for risk and protective factors. METHODS/STUDY POPULATION: Data from 5th, 8th, 9th, and 11th-graders were obtained from the 2019 Minnesota Student Survey (N=96,820). Linear regressions examined the impact of parent connectedness on PA across 4 groups (neither asthma nor obesity [OB], asthma only, OB only, comorbid asthma/OB). The p-value for significance was set at p<.001. For PA, youth reported how many days they were physically active (≥60 min/day) in the last week. Two items assessing youth perception of parent care and ability to talk to parents about their problems were used to measure parent connectedness. BMI was calculated using self-report height/weight, age, and gender. Control variables included age, race/ethnicity, and free/reduced lunch eligibility. Models 2-4 retained parent connectedness variables and added risk and protective factors. RESULTS/ANTICIPATED RESULTS: In Model 1, both parent variables significantly predicted PA for each risk group (β ranges: parent care=.07-.09; parent talk=.04-.05, p<.001), except for the asthma/OB group (parent talk: p>.001). Models 2 and 3 added risk factors. Depression was the most salient risk factor, particularly for the highest risk group (asthma/OB; β =-.13, p<.001). Safe neighborhood was positively associated with PA for all groups (βs= .05, p<.001) except the asthma/OB group (p>.001). In Model 4, extracurricular activity involvement (protective factor) was positively associated with PA across all groups (β ranges=: .07-.11, p<.001), and depression remained significant across all groups (β ranges=-.11 to -.14, p<.001). For models 2-4, only parent care remained significant for the neither asthma nor OB group (β =.04, p<.001). DISCUSSION/SIGNIFICANCE OF FINDINGS: Results demonstrate that although parent care is an important protective factor for youth PA engagement, it is less impactful when additional risk factors (e.g., depression) are present, particularly among the highest risk group (comorbid asthma/OB). Thus, clinical support is needed in addition to parent support among higher risk youth.

